# Reconsidering the structure of the questionnaire for eudaimonic well-being using wide age-range Japanese adult sample: An exploratory analysis

**DOI:** 10.1186/s40359-021-00707-2

**Published:** 2022-01-04

**Authors:** Yu Ishii, Ryota Sakakibara, Aiko Komoto Kubota, Kazuhiro Yamaguchi

**Affiliations:** 1 Graduate School of Education, The University of Tokyo, Tokyo, Japan; 2 Department of Humanities, Faculty of Law, Economics and Humanities, Kagoshima University, Korimoto, 1-21-30, Kagoshima City, Kagoshima 890-0065 Japan; 3 Cooperative Faculty of Education, Utsunomiya University, Tochigi, Japan; 4 Division of Psychology, Faculty of Human Science, University of Tsukuba, Ibaraki, Japan

**Keywords:** Questionnaire for eudaimonic well-being, ESEM, Cultural differences, Age

## Abstract

**Background:**

An increasing amount of research is now highlighting the importance of approaching issues of happiness through eudaimonic well-being. However, the literature does not conclusively show a full understanding of the construct of eudaimonic well-being, as previous studies primarily focused on younger samples from Western countries and only a few studies have attempted to explore its psychological construct through exploratory approaches. Therefore, we conducted a survey among a wide range of age groups in Japan to capture the psychological construct of eudaimonic well-being, through an exploratory analytic approach using Questionnaire for Eudaimonic Wellbeing (QEWB).

**Methods:**

A total of 1126 Japanese participants (580 females, 546 males) were included for analysis. Participants were divided into three age groups according to their age, including 10s to 20s (18–29 years), 30s to 40s (30–49 years) and 50s to 60s (50–69 years). After narrowing down the total number of factors by exploratory structural equation modeling (ESEM), we conducted an ESEM and bifactor ESEM with oblique goemin and oblique bi-geomin rotations for choosing and assessing the final model based on the rotated results and its interpretability.

**Results:**

The results of a parallel analysis and goodness-of-fit indices obtained by ESEM indicated that the QEWB consisted of three or more factors. Both a three-to-six factor and bifactor ESEM with oblique goemin rotation showed that three-factor structure for the 30s to 40s and 50s to 60s and four-factor structure for the 10s to 20s should be chosen, respectively. “Deep and Meaningful Engagement,” a factor only relevant to the 10s to 20s may be an expanded version of what original paper called the Intense Involvement in Activities, with more emphasis on the enthusiastic attitude one has towards activities.

**Conclusions:**

The structure of eudaimonic well-being may differ across cultures and ages, thus requiring further investigation in the field.

**Supplementary Information:**

The online version contains supplementary material available at 10.1186/s40359-021-00707-2.

## Background

While most studies on happiness and well-being have conducted their investigations with a focus on hedonia, an increasing amount of research is now highlighting the importance of approaching issues through eudaimonia, which is a quality of life (QOL) concept referring to the development of one’s best potentials and their application in fulfilling personally expressive and self-concordant goals [1–3]. There have been many attempts to capture the psychological construct of eudaimonic well-being, particularly including the widely implemented Questionnaire for Eudaimonic Well-Being (QEWB) [4]. In fact, the QEWB was the first scale specifically aimed at capturing eudaimonic well-being [5]. Based on philosophical and psychological findings, Waterman et al. [4] suggested that eudaimonic well-being consisted of six core conceptual dimensions, including (1) self-discovery, (2) perceived development of one’s best potentials, (3) a sense of purpose and meaning in life, (4) investment of significant effort in pursuit of excellence, (5) intense involvement in activities, and (6) enjoyment of activities as personally expressive.

While the QEWB is considered an advanced scale because it can measure eudaimonic well-being from multiple perspectives, there have been inconsistent findings about its factor structure. After Waterman proposed QEWB in 2010, in the following decade, various attempts have been made to capture the construct of QEWB. First, contrary to the QEWB’s theoretical assumption, Waterman et al. [4] showed that it was a unifactorial scale via confirmatory factor analysis (CFA) with item-parceling strategy. Pointing out the need for an item-level examination, Schutte et al. [6] conducted a survey among university students in South Africa. Their exploratory factor analysis (EFA) suggested that the QEWB was best structured as a three or four factor scale, with results showing item cross-loadings. Areepattamannil and Hashim [7], who also conducted item-level CFA, however, reported that a single-factor model was a good fit for their data obtained from a survey conducted on adolescents in India.

Further considering these potential item cross-loadings as well as the existence of a single overarching construct, a more recent investigation by Fadda et al. [8] adopted previous proposals [9, 10] in their employment of a bifactor exploratory structural equation modeling (ESEM) framework to investigate the QEWB. More specifically, the researchers used data collected from an Italian sample to conduct a bifactor ESEM based on findings from Schutte et al. [6], which indicated that the QEWB consisted of three or four factors. Their results showed that the QEWB contained both G-factor (which reflect general eudaimonic well-being) and S-factors (which reflect specific sub-concepts of eudaimonic well-being). Later, Fadda et al. [11] conducted the same type of analysis on data collected from a sample of university students in Spain, thus finding evidence for either a three factor or bifactor three factor structure. Note that studies proposing multi-factor structure of the questionnaire have proposed the following factors: Sense of Purpose, Purposeful Personal Expressiveness, Effortful Engagement, Engagement in Rewarding Activities, Living from Beliefs [6, 8, 11].

Although there have been many attempts to do so, the literature does not conclusively show a full understanding of the construct of eudaimonic well-being. There are three main reasons for this. First, no studies have investigated the scale’s construct among East Asian sample. To date, numerous claims have been made about cultural differences in the concepts of happiness and well-being, many of which have pointed out substantial differences in Western and Eastern cultures [12, 13]. One of the most significant differences reported concerns the determinants of happiness, which weighs heavily on the cultural belief of self. A good example would be Kitayama, Markus and Kurokawa’s study [14], which depicted how subjective well-being was closely related to interpersonal engagement among Japanese (Eastern) sample whereas subjective well-being was closely related to interpersonal disengagement among the United States (Western) sample. Uchida and Kitayama [15] also reported that while positive experience can arise from both personal achievement and social harmony in both American and Japanese samples, positive hedonic experience appears to be much more closely aligned with personal achievement in the US, while it is much more closely aligned with social harmony in Japan. These findings indicate the overlapping but fundamental differences of the experience of well-being in the two cultures.

As a more general quality, it has been suggested that happiness may be a broader phenomenon in the Eastern cultures compared to Western cultures. It has been reported in many studies that in the Western cultures, happiness is associated with general hedonic qualities whereas in the Eastern cultures it is also associated with non-positive qualities. Uchida and Kitayama [15] report that a substantial proportion of features of happiness generated by Japanese were nonpositive (transcendental reappraisal) or even negative (social disruption), when there were only a few items that matched these definitions within the American data. This is in line with Kan, Karasawa and Kitayama’s study [16] which studied the happiness experienced within people in Japan and presented that positivity is not necessary for Japanese people’s happiness. They argued that because East Asian cultures have historically cultivated the notion of well-being grounded in the realization of “nothingness,” they have learned to appreciate the mere fact of being and living and enjoy the present moment. These studies indicate that well-being and happiness in the Eastern culture may be a broader notion, including the non-positive features, compared to the Western culture.

Joshanloo [17] specifically illustrated differences in both hedonism and eudaimonism between the two cultures, which is in accord with the aforementioned studies, suggesting that while hedonistic happiness is in accord with the core values of Western cultures, it is not equally valued in Eastern cultures. He states that in the Eastern context, positive emotions are considered too temporary to serve as criteria for happiness, while negative emotions are considered to contribute to spiritual development. These studies overall indicate that while there is a significant overlap between Western and Eastern cultures, for Eastern cultures, happiness and well-being may be a broader feature including non-positive features and the notion of “accepting the present,” while also being a more social, interdependent concept. In these regards, Joshanloo [17] advised caution when applying Western eudaimonistic models and measures in Eastern cultures, particularly since the positive qualities advocated by Eastern eudaimonism may fundamentally differ from those recognized by Western cultures. Based on the claim that happiness is essentially of a eudaimonic character in Eastern cultures, the QEWB’s factor structure should specifically be investigated in East Asia, which will help determine its validity and reliability.

Second, previous studies have primarily focused on young people. However, one study conducted in Italy suggested age-based differences in the construct of eudaimonic well-being. More specifically, results suggested that young adults seemed to cultivate eudaimonic well-being by working hard and investing significant effort in difficult activities, while middle-aged adults seemed to place more emphasis on self-knowledge and setting life goals [18]. In this regard, age-based differences in the structure of happiness have been reported since the late twentieth century [19, 20]. A wider range of age groups should be of great use when attempting to more elaborately study the construct of eudaimonic well-being.

Third, most previous studies have taken a confirmative approach when examining the psychometric properties of the QEWB [7]. This may enable researchers to determine whether a proposed factor structure fits the given data, but does not allow for the discovery of a better construct of eudaimonic well-being. As mentioned above, Fadda et al. [8] investigated the QEWB via ESEM and bifactor ESEM. While this may seem to constitute an exploratory approach, the researchers also used target rotation to specify the solutions for their analyses. Target rotation is conducted by setting zero targets for factor loadings on items that do not belong to the scale associated with the factor while giving free status to all other loadings [21]. The analyses were therefore conducted with strong assumptions about the associations between factors and items. A fully exploratory approach is thus needed to establish a valid construct of eudaimonia that is also generalizable across different age groups and cultures.

To address these gaps in the literature, the present study adopted an exploratory analytical approach to reevaluate the structure of the QEWB among a wide range of Japanese sample. We assumed that within Japanese samples, a person who experiences high eudaimonic well-being and thus experience high Sense of Purpose may not necessarily experience high Purposeful Personal Expressiveness. Purposeful Personal Expressiveness consists of items such as “I believe it is important to know how what I’m doing fits with purposes worth pursuing,” having an active connotation, which in way, may not fit the Eastern characteristic; their appreciation of the mere fact of being. Therefore, we assumed that multifactor models may be best fit for Japanese samples.

After obtaining data, participants were divided into three groups according to their ages, including “10s to 20s” (18–29 years), “30s to 40s” (30–49 years) and “50s to 60s” (50–69 years). Data from each group were then performed ESEM in order to determine the number of factors via various goodness-of-fit indices. After narrowing down the total number of factors, we conducted an ESEM and bifactor ESEM with oblique goemin and oblique bi-geomin rotations. We then chose and assessed the final model based on the rotated results and its interpretability. Finally, we examined the relationships between each QEWB factor and other variables (i.e., age, sex, self-esteem, and life satisfaction) for the purpose of obtaining information to modifying items in the future.

## Methods

### Participants and procedures

This study surveyed two samples of participants.^1^ Those in sample 1 were recruited through an email invitation containing information about the survey including informed consent. Reward points were then given to each person that returned a completed questionnaire. More specifically, sample 1 consisted of 1000 Japanese individuals (500 females, 500 males, *M* = 44.93, *SD* = 13.78, range = 20–69) who were members of an online research panel provided by Cross Marketing (https://www.cross-m.co.jp/en/).^2^

Participants in sample 2 were recruited through an undergraduate course at a Japanese university. These individuals were given information about the survey in advance and were asked to participate if they agreed. Specifically, sample 2 consisted of 165 Japanese undergraduate students (91 females, 69 males, 5 unidentified, *M* = 19.52, *SD* = 1.17, range = 18–23).

After removing those with inappropriate responses (i.e., same answers to all QEWB items) and undisclosed ages, data from a total of 1126 participants (96.65%; 580 females, 546 males, *M* = 41.46, *SD* = 15.58, range = 18–69) were included for analysis. This study was approved by Life Science Research Ethics and Safety, the University of Tokyo.

### Measures

#### QEWB

The Japanese version of the QEWB (see Additional file 1: APPENDIX Table 4) was developed via translation and back-translation. First, all items were translated into Japanese by the first and second author, one of whom is a returnee from the United States and spoke fluent English. Then, both a native English and native Japanese speaker translated the QEWB back into English. The first author checked the accuracy of the back-translated items by comparing them with the original items, and at this phase, modified the items translated to Japanese when necessary. The Japanese version of the QEWB consists of 21 items that are answered on a five-point scale ranging from 0 (strongly disagree) to 4 (strongly agree).

#### Self-esteem

The Japanese version of the Rosenberg Self-Esteem Scale [22] was used to examine the relationship between self-esteem and eudaimonic well-being. The scale consists of 10 items that are answered on a four-point scale ranging from 1 (strongly disagree) to 4 (strongly agree). Reliability indices showed adequate internal consistency for use with each age group (10s to 20s: α = .886, 30s to 40s: α = .891, 50s to 60s: α = .914).

#### Life satisfaction

Based on the original Satisfaction With Life Scale (SWLS) developed by Diener et al. [23], the Japanese version of the SWLS [24] was used to measure and examine the relationship between life satisfaction and eudaimonic well-being. The SWLS consists of five items that are answered on a seven-point scale ranging from 1 (strongly disagree) to 7 (strongly agree). Reliability indices showed adequate internal consistency for use with each age group (10s to 20s: α = .869, 30s to 40s: α = .868, 50s to 60s: α = .882).

### Analyses

We first calculated descriptive statistics for each item in each age group, then determined the number of factors. An ESEM was conducted for each age group using oblique goemin rotation with maximum likelihood estimation. Following Waterman et al. [4], one- to six-factor models were examined. We used the Mplus 8 software [25] with maximum likelihood (ML) to estimate both the ESEM and bifactor-ESEM models. Model fit was assessed for each factor structure in each age group with the following indexes: the chi-square model fit test, log-likelihood, Tucker Lewis Index (TLI), AIC, BIC, sample size adjusted versions of the AIC and BIC (AICC; aBIC), root mean square error of approximation (RMSEA) with 90% confidence interval (90%CI), and standardized root mean square residual (SRMR). The chi-square value was employed to test the hypothesis that the current model is correct. If the *p* value is small, we would reject such null hypothesis, which means the model did not fit the data. TLI is an incremental fit index that evaluates the improvement of the current model from null model which assumes no-correlation among variables. Value between 0.90 and 0.95 is required for TLI and any value above 0.95 would be considered good. AIC, BIC, AICC, and aBIC are all under the umbrella of information criterion index family. They were employed as relative comparison among models and among these indices, lower value is better. The most famous index AIC represents predictive aspect of the model. On the other hand, BIC is related to marginal likelihood that is a probability of data under current model assumption. Both AICC are aBIC were modified version of AIC and BIC so as to be appropriate in finite sample size situation. RMSEA and SRMR are famous absolute measures of fit indices. RMSEA is calculated based on the non-centrality parameter, which is an effect size related statistic of the chi-squared test. SRMR is based on the difference between the observed correlation and the correlation based on the model. RMSEA value 0.05 is considered good and SRMR value less than 0.08 is also considered good. Explanation of fit indices employed in Mplus can be found in p19 to 27 of Wang and Wang [26]. (The explanations of the fit indices presented above are based on Kenny [27] and Technical Appendices of Mplus [28].) We then used the ‘fa.parallel’ function in the R psych package to investigate the number of factors, eigenvalues, cumulative explained variance, and suggested factor numbers via parallel analysis [29]. Eigenvalues were calculated via sample correlation matrix and without missing values.

After narrowing down the number of factors via ESEM and bifactor ESEM with oblique goemin and oblique bi-geomin rotations, the final model was chosen based on the rotated results and its interpretability. While we originally planned to examine correlations between the QEWB, self-esteem, and life satisfaction through a latent model based on the final retained solution, considerable differences were found when comparing the model constructs with those from previous studies [6, 8]. For this reason, we instead examined correlations between the QEWB items and other variables (see Additional file 2: Table S1).

## Results

Table 1 shows the means and standard deviations for each age group. Basic demographic information was revealed as follows: 10s to 20s: age range = 18–29, *M* = 23.09, 186 females and 164 males, 30s to 40s: age range = 30–49, *M* = 40.04, 196 females and 191 males, 50s to 60s: age range = 50–69, *M* = 59.33, 198 females and 191 males.

**Table 1:** Means and standard deviations of each item

	Items	10s to 20s (*n* = 348)		30s to 40s (*n* = 387)		50s to 60s (*n* = 389)	
		Mean	*SD*	Mean	*SD*	Mean	*SD*
1	I find I get intensely involved in many of the things I do each day	2.132	1.082	1.899	0.962	1.864	1.025
2	I believe I have discovered who I really am	1.672	1.109	1.646	1.041	1.784	1.057
3	I think it would be ideal if things came easily to me in my life. ®	1.233	1.079	1.274	0.948	1.347	0.966
4	My life is centered around a set of core beliefs that give meaning to my life	1.810	1.065	1.726	0.967	1.851	0.981
5	It is more important that I really enjoy what I do than that other people are impressed by it	2.790	0.986	2.755	0.899	2.835	0.873
6	I believe I know what my best potentials are and I try to develop them whenever possible	1.839	1.127	1.886	1.006	1.907	1.009
7	Other people usually know better what would be good for me to do than I know myself. ®	2.264	1.010	2.403	0.886	2.455	0.923
8	I feel best when I’m doing something worth investing a great deal of effort in	2.319	1.110	1.961	0.958	1.964	1.055
9	I can say that I have found my purpose in life	1.690	1.208	1.548	1.048	1.643	1.123
10	If I did not find what I was doing rewarding for me, I do not think I could continue doing it	2.710	1.079	2.463	0.911	2.509	0.932
11	As yet, I’ve not figured out what to do with my life. ®	1.612	1.230	1.661	1.130	1.835	1.164
12	I can’t understand why some people want to work so hard on the things that they do. ®	2.853	1.062	2.690	0.940	2.769	0.989
13	I believe it is important to know how what I’m doing fits with purposes worth pursuing	2.368	0.988	2.222	0.819	2.324	0.866
14	I usually know what I should do because some actions just feel right to me	2.014	0.974	2.093	0.888	2.201	0.945
15	When I engage in activities that involve my best potentials, I have this sense of really being alive	2.575	1.045	2.346	0.963	2.416	1.041
16	I am confused about what my talents really are. ®	1.661	1.212	1.902	1.033	2.123	1.065
17	I find a lot of the things I do are personally expressive for me	2.256	1.069	2.140	0.936	2.103	0.979
18	It is important to me that I feel fulfilled by the activities that I engage in	2.899	0.963	2.584	0.913	2.704	0.878
19	If something is really difficult, it probably isn’t worth doing. ®	2.647	0.941	2.336	0.843	2.458	0.868
20	I find it hard to get really invested in the things that I do. ®	2.345	1.066	2.202	1.006	2.383	1.008
21	I believe I know what I was meant to do in life	1.664	1.065	1.716	1.019	1.864	1.072

We then determined the number of factors by calculating eigenvalues for each factor, examining the scree plot, and conducting a parallel analysis. This indicated that three factors should be extracted for the 30s to 40s and 50s to 60s, but that four factors should be extracted for the 10s to 20s. We also conducted an ESEM for each age group, using oblique goemin rotations with maximum likelihood estimations, thus examining one- to six factor models. See Table 2 for the results.

**Table 2:** Goodness-of-fit indices for the estimated models

Group	Factors	Parameters	χ^2^	DF	*p*	Log-likelihood	TLI	AIC	AICC	BIC	aBIC	RMSEA	90%CI	SRMR	Eigen values	Cumulative variance (%)	Parallel analysis
													[Lower, Upper]				
10s to 20s	1	63	982.783	189	< .001	− 9955.233	0.659	20,036.467	20,064.762	20,279.336	20,079.479	.110	[.103, .117]	.100	6.389	30.424	
	2	83	492.496	169	< .001	− 9710.090	0.845	19,586.180	19,638.799	19,906.151	19,642.847	.074	[.067, .082]	.057	2.597	42.790	
	3	102	259.483	150	< .001	− 9593.583	0.941	19,391.167	**19,476.582**	**19,784.384**	*19,460.806*	.046	[.036, .055]	.034	1.670	50.741	
	4	120	187.208	132	.001	− 9557.446	0.966	19,354.892	*19,482.260*	*19,817.500*	**19,436.819**	.035	[.022, .046]	.025	1.232	56.606	✓
	5	137	148.486	115	.019	− 9538.085	0.976	19,350.170	19,529.374	19,878.315	19,443.704	.029	[.012, .041]	.022	0.870	60.750	
	6	153	118.051	99	.093	− 9522.867	0.984	*19,351.735*	19,593.397	19,941.561	19,456.193	.023	[.000, .038]	.019	0.804	64.580	
30s to 40s	1	63	1080.100	189	< .001	− 10,442.523	0.654	21,011.046	21,036.012	21,260.426	21,060.534	.110	[.104, .117]	.104	6.278	29.897	
	2	83	537.562	169	< .001	− 10,171.254	0.840	20,508.508	20,554.528	20,837.057	20,573.707	.075	[.068, .082]	.060	2.676	42.639	
	3	102	280.285	150	< .001	− 10,042.615	0.936	20,289.231	*20,363.217*	**20,692.990**	20,369.354	.047	[.039, .056]	.033	1.761	51.027	
	4	120	187.275	132	.001	− 9996.110	0.969	20,232.220	**20,341.393**	*20,707.231*	20,326.483	.033	[.021, .043]	.024	1.133	56.423	
	5	137	138.828	115	.065	− 9971.887	0.985	*20,217.774*	20,369.629	20,760.078	*20,325.391*	.023	[.000, .036]	.020	0.976	61.070	✓
	6	153	90.856	99	.708	− 9947.901	1.006	**20,201.802**	20,404.051	20,807.441	**20,321.987**	.000	[.000, .021]	.016	0.797	64.865	
50s to 60s	1	63	892.974	189	< .001	− 10,127.432	0.710	20,380.864	20,405.676	20,630.569	20,430.675	.098	[.091, .104]	.086	6.409	30.520	
	2	83	506.902	169	< .001	− 9934.396	0.844	20,034.792	20,080.510	20,363.769	20,100.416	.072	[.065, .079]	.056	2.125	40.640	
	3	102	294.227	150	< .001	− 9828.058	0.925	19,860.117	**19,933.586**	**20,264.402**	19,940.763	.050	[.041, .058]	.034	1.843	49.414	
	4	120	233.434	132	< .001	− 9797.662	0.940	19,835.324	*19,943.682*	*20,310.953*	19,930.202	.044	[.035, .054]	.029	1.142	54.853	✓
	5	137	171.361	115	< .001	− 9766.625	0.962	*19,807.251*	19,957.896	20,350.261	*19,915.570*	.035	[.024, .046]	.024	0.939	59.323	
	6	153	122.342	99	.056	− 9742.116	0.982	**19,790.232**	19,990.760	20,396.660	**19,911.202**	.025	[.000, .038]	.019	0.867	63.450	

Bold indicates the most fitted model, and italic indicates the second fitted model

The goodness-of-fit results were inconsistent for all age groups. However, most indices suggested that models with three or more factors were most appropriate. For the 10s to 20s, the AICC, BIC, and aBIC results suggested between three and four factors, while the parallel analysis suggested four, and the AIC suggested between five and six. For both the 30s to 40s and 50s to 60s, the AICC and BIC results suggested between three and four factors, while the AIC and aBIC suggested between five and six.

Following these results, we conducted three- to six-factor ESEM and bifactor ESEM (2 specific factors + 1 global factor to 5 specific factors + 1 global factor) and examined the factor loadings to determine the best interpretable model. Based on the factor loadings for each item (particularly focusing on those with loadings over .40), we chose a four-factor structure ESEM model with oblique goemin rotation for the 10s to 20s, while three-factor structure ESEM models with oblique goemin rotations were chosen for the 30s to 40s and 50s to 60s (Table 3; Additional file 3: APPENDIX Table 5 to 7). Other models failed to produce interpretable solutions, including the factor structures with bifactor rotations. For example, some factors had none or only one or two items with factor loadings above .40.

**Table 3:** Standardized factor loadings and residual variances

10s to 20s						30s to 40s					50s to 60s				
Items	F1	F2	F3	F4	Residual variance	Items	F1	F2	F3	Residual variance	Items	F1	F2	F3	Residual variance
1	**.687**	.100	− .129	.026	.477	21	**.814**	.013	.019	.329	11	**.854**	.111	− .221	.332
17	**.611**	.093	.092	.069	.493	9	**.793**	.013	− .042	.368	21	**.800**	.035	− .045	.639
8	**.609**	.009	.158	.101	.493	11	**.772**	− .153	.262	.331	9	**.799**	− .149	.026	.355
4	**.539**	.337	.031	− .018	.435	4	**.748**	.065	− .104	.408	2	**.772**	− .060	− .008	.419
7	**− .516**	.210	.062	.232	.805	2	**.744**	.009	− .047	.446	4	**.660**	− .120	.069	.528
6	**.467**	.391	.007	− .017	.474	6	**.673**	.192	− .015	.442	16	**.632**	.308	− .235	.497
15	**.430**	− .022	.379	.069	.562	16	**.535**	− .264	.393	.497	6	**.601**	− .020	.215	.487
14	**.413**	.360	.127	.024	.516	14	**.492**	.268	.176	.591	14	**.543**	.126	.158	.579
11	.012	**.651**	− .210	.212	.396	1	**.489**	.172	− .099	.678	20	**.445**	.337	.020	.639
9	.249	**.629**	.037	.012	.394	17	**.468**	.334	.029	.587	1	**.441**	− .016	.139	.736
16	− .048	**.585**	− .278	.136	.523	3	.345	− .306	− .007	.844	7	− .021	**.623**	− .044	.605
21	.362	**.573**	− .035	− .102	.402	18	.016	**.676**	.218	.544	19	− .006	**.453**	.148	.792
2	.293	**.535**	.000	− .033	.503	13	.106	**.615**	− .046	.564	12	.083	**.444**	.352	.680
10	.057	− .017	**.504**	.002	.725	15	.220	**.609**	.016	.512	18	.012	.228	**.731**	.451
5	.011	.136	**.486**	.095	.735	10	− .098	**.553**	− .071	.692	13	− .024	.002	**.551**	.707
3	.261	− .027	**− .460**	.066	.780	8	.270	**.455**	− .064	.643	15	.211	.065	**.535**	.577
18	**.427**	− .037	**.447**	.260	.387	5	− .094	**.450**	.126	.818	10	− .111	.052	**.473**	.813
13	.246	.138	**.429**	− .086	.664	12	.004	.319	**.664**	.535	8	.241	− .099	**.434**	.652
12	− .025	.046	.042	**.820**	.322	19	− .045	.196	**.519**	.736	17	.395	− .049	**.409**	.541
19	.052	− .145	− .062	**.645**	.587	20	**.418**	.002	**.454**	.591	5	− .044	.326	.376	.802
20	.163	.070	− .341	**.423**	.627	7	− .176	.017	.344	.863	3	.237	− .035	− .248	.935
F2	.443					F2	.265				F2	.146			
F3	.288	− .060				F3	.071	− .185			F3	.415	− .136		
F4	.332	.239	− .020												

Factor loadings over 0.40 appear in bold

For each age groups, there seemed to be three similar factors that were also reported in Fadda [6] and Schutte et al. [8]: ‘Sense of purpose,’ ‘Purposeful Personal Expressiveness,’ and ‘Effortful Engagement.’ Sense of Purpose factor (10s to 20s F2, 30s to 40s F1, 50s to 60s F1) contained items such as ‘I believe I know what I was meant to do in life (item 21)’ and ‘As yet, I’ve not figured out what to do with my life (item 11; reverse scored),’ while the Purposeful Personal Expressiveness factor (10s to 20s F3, 30s to 40s F2, 50s to 60s F3) contained items such as ‘It is more important that I really enjoy what I do than that other people are impressed by it (item 18)’ and ‘If I did not find what I was doing rewarding for me, I do not think I could continue doing it (item 10).’ Finally, the Effortful Engagement factor (10s to 20s F4, 30s to 40s F3, 50s to 60s F2) contained items such as ‘I can’t understand why some people want to work so hard on the things that they do (item 12; reverse scored)’ and ‘If something is really difficult, it probably isn’t worth doing (item 19; reverse scored).’

As mentioned, results for the 10s to 20s suggested an additional factor (F1), which we named ‘Deep and Meaningful Engagement.’ This factor contained items such as ‘I find I get intensely involved in many of the things I do each day (item 1),’ ‘I find a lot of the things I do are personally expressive for me (item 17),’ and ‘When I engage in activities that involve my best potentials, I have this sense of really being alive (item 15).’ It is important to note that this factor comprehensively included all six categories proposed by Waterman et al. [4], and is the only factor that was moderately correlated with other factors within the 10s to 20s.

## Discussion

The present study was aimed to reconsider the structure of the QEWB with a wide range of age in Japanese sample using an exploratory approach. Based on goodness-of-fit indices and interpretability, we found a higher possibility that the scale should consist of not one or six factors, but actually consists with three to five factors. More specifically, our results suggested that a four-factor structure was most appropriate for individuals aged between 18 and 29 years, while a three-factor structure was best for individuals ranging between 30–49 and 50–69.

Regarding the original QEWB, Waterman et al. [4] first presumed the existence of six interrelated categories, including (1) self-discovery, (2) perceived development one’s best potentials, (3) a sense of purpose and meaning in life, (4) investment of significant effort in pursuit of excellence, (5) intense involvement in activities, and (6) enjoyment of activities as personally expressive. Further, the researchers conducted a pilot study indicating that the scale was unidimensional. This was later disputed, however. Primarily based on goodness-of-fit indices, Fadda et al. [8, 11] claimed that either a three-factor or bifactor three-factor structure may be best in terms of understanding the QEWB. Note that the fit of bifactor three factor model is the same as four factor ESEM model because the two models would show exactly the same likelihood. This emphasizes the need to consider interpretability when determining the appropriateness of bifactor structures. Contrary to the previous studies listed above, this study found that a four-factor model was best for the 10s to 20s, while a three-factor model was best for both the 30s to 40s and 50s to 60s. These results may enable a better interpretation of the QEWB. Indeed, our results are congruent with Schutte et al. [6], who also reported that three or four factors would better explain the data (Additional file 2).

Despite Joshanloo’s [17] warnings about the use of Western eudaimonistic models and measures in Eastern cultures, the results of this study, Schutte et al. [6], and Fadda et al. [8, 11] suggest that Sense of Purpose and Effortful Engagement consistently appear in the QEWB across cultures and age groups. While perhaps less robust, Purposeful Personal Expressiveness was also supported in these studies. Following this evidence, all three factors may be central aspects of eudaimonic well-being, which therefore robustly appear in almost every study.

In this study, Deep and Meaningful Engagement was newly presented in the 10s to 20s. Notably, this factor included all six categories originally mentioned in Waterman et al. [4]. It was also moderately correlated with the other factors, thus indicating a more general aspect of eudaimonic well-being. Nevertheless, the highest factor loading (0.687) was found for the item ‘I find I get intensely involved in many of the things I do each day,’ which may especially be reflected by and/or serve as an expanded version of what Waterman et al. [4] (2010) called the Intense Involvement in Activities category. While Waterman et al. [4] particularly focused on the intensity of one’s commitment to activities when describing this category, this factor implies more that the activities are personally expressive (item 17), feel right for one to engage in (item 14), and involve one’s best potentials (item 15). Thus, while Effortful Engagement factor reflects the willingness to put effort into matters regardless of difficulty [6], this factor weighs more on the ‘flow’ and enthusiastic attitude one may have toward those activities.

Deep and Meaningful Engagement may have only appeared in the 10s to 20s due to variations in how people of different ages experience the QEWB factors. First, it must be noted that the items included in Deep and Meaningful Engagement did not dissolve in the 30s to 40s and 50s to 60s, but were instead included in other factors (e.g., Sense of Purpose). Second, although we mentioned above that Sense of Purpose, Effortful Engagement, and Purposeful Personal Expressiveness appeared in all age groups, we cannot disregard the fact that there were intergroup differences between the items included in each factor. For example, Sense of Purpose only contained five items in the 10s to 20s, but contained 11 and 10 in the 30s to 40s and 50s to 60s, respectively. Further, many of the items included in Deep and Meaningful Engagement were also found in Sense of Purpose in the 30s to 40s and 50s to 60s. Although all respondents aged 20–69 seemed to experience similar eudaimonic aspects, these results may indicate differences in the way they are experienced. In other words, older people may have a broader scope of Sense of Purpose than younger people, thus creating a new, broader factor in the 10s to 20s.

In addition, results may heavily reflect the statistical methods and scale development procedures used in this and previous studies. In fact, we reported the factor structure results obtained from rotations aimed for a simple structure. However, the complexity of item wordings resulted in many multiple factor loadings, which prevented us from reporting simple structures. Schutte et al. [6] also pointed out that Effortful Engagement factor may be the result of a methodological issue, since all items included in the factor were reverse-phrased. Further research is therefore needed to determine the most appropriate methodology for examining the concept of eudaimonia.

It is also important to remember that the goodness-of-fit indices were inconsistent, which may suggest the need to reconsider the scale at the item level. In other words, we should reevaluate whether the 21 scale items are fully adequate for capturing the concept of eudaimonic well-being. For example, as Waterman et al. [4] claimed, ‘the process of self-discovery may be central to eudaimonic functioning.’ However, when looking at items that should reflect this idea (e.g., ‘I can say that I have found my purpose in life’ and ‘I believe I know what I was meant to do in life’), self-discovery is not depicted as a ‘process,’ but as a ‘completed’ state. As being in the process of finding one’s purpose and potentials in life is central to this concept, it seems necessary to include items that reflect it.

In terms of future research on scale development, we should note that while we did not produce evidence of cultural differences in how eudaimonic well-being is perceived, it would be premature to conclude that none exist. For example, the QEWB does not include items that reflect fulfillment through interpersonal relationships, despite this being an important aspect of happiness in East Asia. Also, the more negative aspect of well-being [15] and mere appreciation of being [16] should may be reflected in the items. It may therefore be necessary to reexamine the concept from an Eastern eudaimonic perspective [17]. This should entail not only the comparison between Western and Eastern viewpoints, but discussion on what the essence of eudaimonic well-being is, regardless of cultural differences.

Taking these perspectives into account, additional research may be required to develop a scale that substantially reflects the concept of eudaimonic well-being, particularly one that is not a methodological artifact and contains items of less complexity.

### Limitations and future perspectives

This study has several limitations. First, we did not fully examine the relationship between the QEWB and external variables, more specifically in regard to convergent validity. Additional research should therefore investigate this point. Secondly, although it was not a key objective to investigate developmental changes in the experience of eudaimonic well-being, it should be noted that this study employed a cross-sectional design. Considering the wide variation in participant ages, future studies should also examine developmental changes in the experience of eudaimonic well-being. This developmental change mentioned here is not just limited to the change of the scale score value on common scale but to incorporate change of the construct of eudaimonic well-being. Our study indicated such construct level difference across age groups should be reconsidered.

Despite these limitations, this was the first study to investigate the structure of the QEWB among a wide age range of Japanese sample. It was also the first to consider Deep and Meaningful Engagement. Our findings are particularly valuable because we adopted an exploratory approach to examine the appropriateness of the one-to-six factor structure originally suggested by Waterman et al. [4], thus eliminating the potential biases of the researchers. However, continued research is needed to further investigate the concept of eudaimonic well-being from both the theoretical and empirical perspectives.

One future direction from the data analysis perspective may be to conduct multiple group analysis. This study aimed to re-consider the structure of QEWB questionnaire for each age group and the data analysis was separately conducted for this purpose. To test equivalency of factor loading across age group, multiple group SEM analysis is required. Another possibility of data analysis is combining all age group as one sample and conducting EFA, which we did not do in this study due to different research purpose. This might provide a clue of a general eudaimonic well-being scale across age group. If such general scale could be established, the quantitative developmental change could be assessed. We opened the data employed in this study to allow re-analysis to be freely conducted.

## Supplementary Information

**Additional file 1:** The Japanese version of the QEWB.

**Additional file 2:** The correlations and confidence intervals between QEWB items and the variables of sex, age, self-esteem, and life satisfaction.

**Additional file 3:** The results of ESEM with the items and the factor labels added.

## Data Availability

The datasets supporting the conclusions of this article are available in the Open Science Framework, [https://osf.io/62daf/].

## Abbreviations

QEWB: Questionnaire for eudaimonic well-being
SWLS: Satisfaction With Life Scale
QOL: Quality of life
ESEM: Exploratory structural equation modeling
CFA: Confirmatory factor analysis
EFA: Exploratory factor analysis
ML: Maximum likelihood
TLI: Tucker Lewis Index
AICC: The corrected Akaike’s information criterion
aBIC: Akaike’s Bayesian information criterion
RMSEA: Root mean square error of approximation
90%CI: 90% confidence interval
SRMR: Standardized root mean square residual

## Footnotes

1 We obtained data from the university students (*N* = 106, 22 females, 80 males, 4 unidentified, *M* = 20.05, *SD* = 2.05, range = 19–36) prior to the main survey, thus confirming that the translated items were correctly understood. We also checked for problems related to the distributions of each item score.

2 We also conducted a web survey among 500 participants who were members of another online research company. However, their data were not used in the analysis due to very poor quality; for example, about 20% of participants delivered the same responses to different items. The reliability of self-esteem was also very low (α = .25).
