# Supplementary material for: Reconsidering the structure of the questionnaire for eudaimonic well-being using wide age-range Japanese adult sample: An exploratory analysis
Source: BMC Psychol. 2022 Jan 4;10:3. doi: 10.1186/s40359-021-00707-2 (PMC8729131; doi:10.1186/s40359-021-00707-2)
Supplement: Supplementary file 1 — Additional file 1: The Japanese version of the QEWB. [file 40359_2021_707_MOESM1_ESM.docx]

**Additional file 1**

APPENDIX Table 4. The Japanese version of the QEWB.

| Items | |
| --- | --- |
| 1 | 私は、毎日行なっている活動の多くに没頭していると思う |
| 2 | 私は、本当の自分を見つけられたと思う |
| 3 | もし自分の人生においていろいろなことが楽に済めば、それは理想的なことだと思う（逆転項目） |
| 4 | 私の人生は、人生に意味をもたらすしっかりとした信念を軸に、展開している |
| 5 | 他の人が感心するかどうかよりも、自分のしていることを本当に楽しむことの方が大切である |
| 6 | 私は、自分の一番の強みは何であるかを知っているし、可能な限りそれを伸ばそうと努力していると思う |
| 7 | 私にとって何が良いのかについて、私自身よりも他の人がよく知っていることが多い（逆転項目） |
| 8 | 私は、多大な努力を投資する価値のあることをしているとき、最高の気分になる |
| 9 | 私は、人生の目標を見つけたと言うことができる |
| 10 | もし自分が取り組んでいることにやりがいを見出せなかったら、それを続けられないと思う |
| 11 | いまだに私は人生で何をすべきか見つけ出せていない（逆転項目） |
| 12 | なぜ自身の活動を頑張りたいと思う人がいるのか、理解できない（逆転項目） |
| 13 | 自分のしていることが、追求する価値のある目的に沿っているものであるのか、それを知ることが重要だと思う |
| 14 | いくつかの行動は自分にとってしっくりくるため、たいてい自分が何をすべきかわかっている |
| 15 | 自分の一番の強みを活かせる活動に取り組んでいるとき、自分が確かに生きているという気持ちになる |
| 16 | 何が自分の本当の才能であるのかわからず、困惑している（逆転項目） |
| 17 | 私が取り組んでいるたくさんのことは、私自身を表現するものだと思う |
| 18 | 私にとって、自身が取り組んでいる活動によって充実感を得ることは、大切なことである |
| 19 | もし何かがとても困難なものであるとき、それはおそらく取り組む価値がないものである（逆転項目） |
| 20 | 私は、自分が行っていることに全力で取り組むことができない（逆転項目） |
| 21 | 私は、人生の中で何をすべきなのか、わかっていると思う |
| Items range from 0（全く当てはまらない） to 4（とても当てはまる）. | |
