# Supplementary material for: Reconsidering the structure of the questionnaire for eudaimonic well-being using wide age-range Japanese adult sample: An exploratory analysis
Source: BMC Psychol. 2022 Jan 4;10:3. doi: 10.1186/s40359-021-00707-2 (PMC8729131; doi:10.1186/s40359-021-00707-2)
Supplement: Supplementary file 2 — Additional file 2: The correlations and confidence intervals between QEWB items and the variables of sex, age, self-esteem, and life satisfaction. [file 40359_2021_707_MOESM2_ESM.docx]

**Additional file 2**

Table S1. Correlations between each item and other variables (95% confidence intervals).

|  |  | Sex | | | | | | Age | | | | | | Self-esteem | | | | | | Life satisfaction | | | | | |
| --- | --- | --- | --- | --- | --- | --- | --- | --- | --- | --- | --- | --- | --- | --- | --- | --- | --- | --- | --- | --- | --- | --- | --- | --- | --- |
| Group | Item | *r* |  | 95%CI | | |  | *r* |  | 95%CI | | |  | *r* |  | 95%CI | | |  | *r* |  | 95%CI | | |  |
|  |  |  | [ | Lower | , | Upper | ] |  | [ | Lower | , | Upper | ] |  | [ | Lower | , | Upper | ] |  | [ | Lower | , | Upper | ] |
| 10s to 20s | 1 | .027 | [ | -.061 | , | .115 | ] | -.360 | [ | -.437 | , | -.284 | ] | .444 | [ | .373 | , | .514 | ] | .478 | [ | .411 | , | .546 | ] |
|  | 2 | -.052 | [ | -.140 | , | .036 | ] | -.195 | [ | -.280 | , | -.111 | ] | .540 | [ | .478 | , | .602 | ] | .565 | [ | .505 | , | .625 | ] |
|  | 3 | -.047 | [ | -.135 | , | .041 | ] | .058 | [ | -.029 | , | .146 | ] | .155 | [ | .069 | , | .241 | ] | .109 | [ | .022 | , | .196 | ] |
|  | 4 | .000 | [ | -.088 | , | .088 | ] | -.189 | [ | -.274 | , | -.104 | ] | .541 | [ | .478 | , | .603 | ] | .517 | [ | .452 | , | .582 | ] |
|  | 5 | .013 | [ | -.075 | , | .101 | ] | -.088 | [ | -.175 | , | .000 | ] | .167 | [ | .082 | , | .253 | ] | .197 | [ | .112 | , | .281 | ] |
|  | 6 | .000 | [ | -.088 | , | .088 | ] | -.212 | [ | -.296 | , | -.128 | ] | .569 | [ | .510 | , | .629 | ] | .483 | [ | .416 | , | .551 | ] |
|  | 7 | .090 | [ | .003 | , | .178 | ] | .141 | [ | .055 | , | .227 | ] | -.013 | [ | -.101 | , | .075 | ] | -.133 | [ | -.219 | , | -.046 | ] |
|  | 8 | -.034 | [ | -.121 | , | .054 | ] | -.337 | [ | -.415 | , | -.259 | ] | .312 | [ | .233 | , | .392 | ] | .320 | [ | .241 | , | .399 | ] |
|  | 9 | .022 | [ | -.066 | , | .110 | ] | -.151 | [ | -.237 | , | -.065 | ] | .481 | [ | .413 | , | .548 | ] | .459 | [ | .389 | , | .528 | ] |
|  | 10 | .009 | [ | -.079 | , | .097 | ] | -.146 | [ | -.232 | , | -.060 | ] | -.098 | [ | -.185 | , | -.011 | ] | -.063 | [ | -.151 | , | .024 | ] |
|  | 11 | -.012 | [ | -.100 | , | .076 | ] | -.096 | [ | -.183 | , | -.009 | ] | .540 | [ | .477 | , | .602 | ] | .478 | [ | .410 | , | .545 | ] |
|  | 12 | -.060 | [ | -.147 | , | .028 | ] | -.222 | [ | -.306 | , | -.139 | ] | .285 | [ | .204 | , | .366 | ] | .249 | [ | .166 | , | .331 | ] |
|  | 13 | .021 | [ | -.067 | , | .109 | ] | -.183 | [ | -.268 | , | -.098 | ] | .131 | [ | .045 | , | .218 | ] | .186 | [ | .101 | , | .270 | ] |
|  | 14 | -.078 | [ | -.166 | , | .009 | ] | -.212 | [ | -.296 | , | -.128 | ] | .480 | [ | .413 | , | .548 | ] | .443 | [ | .372 | , | .513 | ] |
|  | 15 | -.083 | [ | -.170 | , | .004 | ] | -.249 | [ | -.332 | , | -.167 | ] | .171 | [ | .086 | , | .256 | ] | .243 | [ | .161 | , | .326 | ] |
|  | 16 | .044 | [ | -.044 | , | .132 | ] | .012 | [ | -.076 | , | .100 | ] | .505 | [ | .439 | , | .570 | ] | .373 | [ | .297 | , | .448 | ] |
|  | 17 | -.092 | [ | -.179 | , | -.004 | ] | -.256 | [ | -.338 | , | -.173 | ] | .451 | [ | .381 | , | .521 | ] | .434 | [ | .363 | , | .506 | ] |
|  | 18 | -.149 | [ | -.235 | , | -.064 | ] | -.343 | [ | -.421 | , | -.266 | ] | .276 | [ | .195 | , | .358 | ] | .327 | [ | .249 | , | .406 | ] |
|  | 19 | -.050 | [ | -.138 | , | .037 | ] | -.227 | [ | -.310 | , | -.143 | ] | .199 | [ | .115 | , | .283 | ] | .153 | [ | .067 | , | .239 | ] |
|  | 20 | .037 | [ | -.051 | , | .125 | ] | -.091 | [ | -.179 | , | -.004 | ] | .384 | [ | .309 | , | .459 | ] | .298 | [ | .218 | , | .379 | ] |
|  | 21 | -.074 | [ | -.161 | , | .014 | ] | -.151 | [ | -.237 | , | -.065 | ] | .467 | [ | .398 | , | .536 | ] | .470 | [ | .402 | , | .539 | ] |

Table S1

(*continued*)

|  |  | Sex | | | | | | Age | | | | | | Self-esteem | | | | | | Life satisfaction | | | | | |
| --- | --- | --- | --- | --- | --- | --- | --- | --- | --- | --- | --- | --- | --- | --- | --- | --- | --- | --- | --- | --- | --- | --- | --- | --- | --- |
| Group | Item | *r* |  | 95%CI | | |  | *r* |  | 95%CI | | |  | *r* |  | 95%CI | | |  | *r* |  | 95%CI | | |  |
|  |  |  | [ | Lower | , | Upper | ] |  | [ | Lower | , | Upper | ] |  | [ | Lower | , | Upper | ] |  | [ | Lower | , | Upper | ] |
| 30s to 40s | 1 | .135 | [ | .053 | , | .217 | ] | .033 | [ | -.050 | , | .117 | ] | .380 | [ | .308 | , | .451 | ] | .368 | [ | .295 | , | .440 | ] |
|  | 2 | -.076 | [ | -.159 | , | .007 | ] | .074 | [ | -.009 | , | .157 | ] | .547 | [ | .488 | , | .605 | ] | .520 | [ | .459 | , | .581 | ] |
|  | 3 | -.041 | [ | -.125 | , | .042 | ] | -.015 | [ | -.098 | , | .069 | ] | .216 | [ | .137 | , | .296 | ] | .134 | [ | .052 | , | .217 | ] |
|  | 4 | .019 | [ | -.065 | , | .102 | ] | .108 | [ | .025 | , | .191 | ] | .475 | [ | .410 | , | .540 | ] | .504 | [ | .441 | , | .566 | ] |
|  | 5 | -.027 | [ | -.110 | , | .057 | ] | .011 | [ | -.072 | , | .095 | ] | .054 | [ | -.029 | , | .137 | ] | .083 | [ | .000 | , | .166 | ] |
|  | 6 | .086 | [ | .003 | , | .169 | ] | .035 | [ | -.048 | , | .119 | ] | .485 | [ | .421 | , | .549 | ] | .431 | [ | .363 | , | .499 | ] |
|  | 7 | .044 | [ | -.039 | , | .128 | ] | .064 | [ | -.020 | , | .147 | ] | .003 | [ | -.081 | , | .087 | ] | -.099 | [ | -.182 | , | -.016 | ] |
|  | 8 | .118 | [ | .036 | , | .200 | ] | .006 | [ | -.077 | , | .090 | ] | .240 | [ | .161 | , | .319 | ] | .199 | [ | .118 | , | .279 | ] |
|  | 9 | -.034 | [ | -.117 | , | .050 | ] | .043 | [ | -.041 | , | .126 | ] | .502 | [ | .440 | , | .565 | ] | .493 | [ | .430 | , | .556 | ] |
|  | 10 | .029 | [ | -.055 | , | .112 | ] | -.098 | [ | -.181 | , | -.016 | ] | .030 | [ | -.054 | , | .114 | ] | -.006 | [ | -.089 | , | .078 | ] |
|  | 11 | -.025 | [ | -.109 | , | .059 | ] | .082 | [ | -.001 | , | .165 | ] | .511 | [ | .449 | , | .573 | ] | .492 | [ | .429 | , | .556 | ] |
|  | 12 | -.105 | [ | -.188 | , | -.023 | ] | -.023 | [ | -.107 | , | .060 | ] | .151 | [ | .069 | , | .233 | ] | .198 | [ | .118 | , | .278 | ] |
|  | 13 | .007 | [ | -.077 | , | .090 | ] | -.011 | [ | -.095 | , | .072 | ] | .116 | [ | .033 | , | .198 | ] | .206 | [ | .126 | , | .287 | ] |
|  | 14 | .034 | [ | -.050 | , | .117 | ] | -.016 | [ | -.099 | , | .068 | ] | .424 | [ | .355 | , | .492 | ] | .367 | [ | .294 | , | .439 | ] |
|  | 15 | .021 | [ | -.062 | , | .105 | ] | .026 | [ | -.058 | , | .110 | ] | .251 | [ | .172 | , | .329 | ] | .244 | [ | .166 | , | .323 | ] |
|  | 16 | -.017 | [ | -.101 | , | .066 | ] | .100 | [ | .017 | , | .182 | ] | .442 | [ | .374 | , | .509 | ] | .370 | [ | .298 | , | .442 | ] |
|  | 17 | .011 | [ | -.072 | , | .095 | ] | .085 | [ | .002 | , | .168 | ] | .308 | [ | .232 | , | .383 | ] | .305 | [ | .229 | , | .381 | ] |
|  | 18 | .065 | [ | -.018 | , | .148 | ] | .037 | [ | -.047 | , | .120 | ] | .163 | [ | .081 | , | .244 | ] | .145 | [ | .064 | , | .227 | ] |
|  | 19 | -.021 | [ | -.105 | , | .062 | ] | .008 | [ | -.075 | , | .092 | ] | .153 | [ | .071 | , | .234 | ] | .071 | [ | -.012 | , | .154 | ] |
|  | 20 | -.023 | [ | -.107 | , | .060 | ] | .051 | [ | -.032 | , | .135 | ] | .432 | [ | .364 | , | .500 | ] | .323 | [ | .248 | , | .398 | ] |
|  | 21 | -.014 | [ | -.098 | , | .070 | ] | .063 | [ | -.021 | , | .146 | ] | .581 | [ | .526 | , | .637 | ] | .504 | [ | .441 | , | .566 | ] |

Table S1

(*continued*)

|  |  | Sex | | | | | | Age | | | | | | Self-esteem | | | | | | Life satisfaction | | | | | |
| --- | --- | --- | --- | --- | --- | --- | --- | --- | --- | --- | --- | --- | --- | --- | --- | --- | --- | --- | --- | --- | --- | --- | --- | --- | --- |
| Group | Item | *r* |  | 95%CI | | |  | *r* |  | 95%CI | | |  | *r* |  | 95%CI | | |  | *r* |  | 95%CI | | |  |
|  |  |  | [ | Lower | , | Upper | ] |  | [ | Lower | , | Upper | ] |  | [ | Lower | , | Upper | ] |  | [ | Lower | , | Upper | ] |
| 50s to 60s | 1 | .121 | [ | .039 | , | .203 | ] | -.025 | [ | -.109 | , | .058 | ] | .301 | [ | .225 | , | .377 | ] | .296 | [ | .220 | , | .372 | ] |
|  | 2 | -.062 | [ | -.145 | , | .021 | ] | .131 | [ | .049 | , | .213 | ] | .485 | [ | .421 | , | .549 | ] | .471 | [ | .406 | , | .536 | ] |
|  | 3 | .070 | [ | -.013 | , | .153 | ] | .092 | [ | .010 | , | .175 | ] | .120 | [ | .038 | , | .202 | ] | .116 | [ | .033 | , | .198 | ] |
|  | 4 | .107 | [ | .025 | , | .190 | ] | .088 | [ | .005 | , | .170 | ] | .414 | [ | .345 | , | .483 | ] | .369 | [ | .297 | , | .441 | ] |
|  | 5 | -.024 | [ | -.107 | , | .060 | ] | .084 | [ | .001 | , | .167 | ] | .133 | [ | .051 | , | .215 | ] | .094 | [ | .011 | , | .177 | ] |
|  | 6 | .049 | [ | -.034 | , | .133 | ] | .029 | [ | -.054 | , | .113 | ] | .468 | [ | .403 | , | .533 | ] | .296 | [ | .220 | , | .372 | ] |
|  | 7 | -.027 | [ | -.110 | , | .057 | ] | .011 | [ | -.072 | , | .095 | ] | .184 | [ | .103 | , | .264 | ] | .028 | [ | -.055 | , | .111 | ] |
|  | 8 | .104 | [ | .022 | , | .187 | ] | .002 | [ | -.082 | , | .085 | ] | .206 | [ | .127 | , | .286 | ] | .103 | [ | .021 | , | .186 | ] |
|  | 9 | .020 | [ | -.064 | , | .103 | ] | .129 | [ | .047 | , | .211 | ] | .458 | [ | .392 | , | .524 | ] | .465 | [ | .400 | , | .530 | ] |
|  | 10 | -.004 | [ | -.087 | , | .079 | ] | .002 | [ | -.082 | , | .085 | ] | -.076 | [ | -.159 | , | .007 | ] | -.087 | [ | -.170 | , | -.004 | ] |
|  | 11 | .030 | [ | -.053 | , | .114 | ] | .191 | [ | .111 | , | .272 | ] | .545 | [ | .487 | , | .604 | ] | .433 | [ | .366 | , | .501 | ] |
|  | 12 | -.096 | [ | -.179 | , | -.014 | ] | .067 | [ | -.016 | , | .150 | ] | .246 | [ | .168 | , | .325 | ] | .147 | [ | .066 | , | .229 | ] |
|  | 13 | .129 | [ | .047 | , | .211 | ] | .067 | [ | -.016 | , | .150 | ] | .060 | [ | -.023 | , | .143 | ] | .045 | [ | -.038 | , | .129 | ] |
|  | 14 | -.053 | [ | -.136 | , | .030 | ] | .041 | [ | -.042 | , | .124 | ] | .422 | [ | .354 | , | .491 | ] | .311 | [ | .236 | , | .387 | ] |
|  | 15 | .097 | [ | .014 | , | .179 | ] | .065 | [ | -.018 | , | .148 | ] | .262 | [ | .184 | , | .340 | ] | .180 | [ | .099 | , | .261 | ] |
|  | 16 | .088 | [ | .006 | , | .171 | ] | .178 | [ | .097 | , | .258 | ] | .563 | [ | .506 | , | .620 | ] | .423 | [ | .354 | , | .491 | ] |
|  | 17 | .009 | [ | -.074 | , | .093 | ] | -.009 | [ | -.092 | , | .075 | ] | .359 | [ | .286 | , | .431 | ] | .301 | [ | .225 | , | .377 | ] |
|  | 18 | .044 | [ | -.040 | , | .127 | ] | .120 | [ | .038 | , | .202 | ] | .230 | [ | .151 | , | .309 | ] | .115 | [ | .033 | , | .197 | ] |
|  | 19 | .051 | [ | -.033 | , | .134 | ] | .019 | [ | -.064 | , | .103 | ] | .178 | [ | .097 | , | .259 | ] | .094 | [ | .011 | , | .176 | ] |
|  | 20 | .103 | [ | .021 | , | .186 | ] | .097 | [ | .014 | , | .179 | ] | .430 | [ | .362 | , | .498 | ] | .338 | [ | .264 | , | .412 | ] |
|  | 21 | -.019 | [ | -.102 | , | .064 | ] | .112 | [ | .029 | , | .194 | ] | .533 | [ | .473 | , | .593 | ] | .421 | [ | .353 | , | .490 | ] |

Table S1 shows the correlations and confidence intervals between QEWB items and the variables of sex, age, self-esteem, and life satisfaction. There were few correlations between each item and sex across all age groups. On the other hand, there were negative relationships between age and several items (i.e., numbers 1, 6, 8, 12, 14, 15, 17, 18, and 19) for the 10s and 20s. While we found no substantial differences between age groups for the relationships with self-esteem and life satisfaction, some items exhibited slightly different tendencies depending on the degree of correlation. For the 10s and 20s, for example, we found larger correlations between item 1 and both self-esteem (*r* = .444) and life satisfaction (r =.478) when compared to those for the 30s to 40s (*r* =.380, 301) and 50s to 60s (*r* =.301, .296) groups. Further, the correlations between item 21 and both self-esteem (*r* =.581) and life satisfaction (*r* =.504) were largest for the 30s and 40s, while those between item 16 and the same two respective variables (*r* =.563, .423) were largest for the 50s and 60s. Overall, the correlations between each item and both self-esteem and life satisfaction tended to be comparatively larger for the 10s and 20s.

These results have important implications for future scale development. For example, some items showed negative correlations with age (*r* < |.20|), but only in the 10s and 20s. This may indicate that scores are more likely to decrease with younger ages. Further, some items (e.g., 1, 16, and 21) showed different correlation patterns with self-esteem and life satisfaction; these are thought to reflect different aspects of eudaimonic well-being found between age groups. For example, item 1 (‘I find I get intensely involved in many of the things I do each day’) showed slightly higher correlations with self-esteem and life satisfaction in the younger group, which implies that working hard especially led to higher eudaiomonic well-being for those individuals. This finding is also congruent with Sotgiu et al. [1].

Several items showed small correlations with self-esteem and life satisfaction. For example, the absolute value of the correlation coefficient for item 10 was smaller than .010 for all age groups. The ability to continue something rewarding is one of the important components of eudaimonia. However, the ability to continue doing something even if it is not rewarding can be thought to reflect "grit," which is thought to increase self-esteem and life satisfaction (e.g., Li et al. [2]). Therefore, high (low) scores on item 10 may reflect a mixture of high (low) eudaimonia and low (high) grit, resulting in a lack of association with self-esteem and life satisfaction. Items 5 and 7 also showed low absolute values of correlation with self-esteem and life satisfaction (*r* < |.20|). These items measure the degree to which a person has established the self as an individual, without depending on relationships with others. However, for Japanese people, happiness and well-being have been shown to be highly dependent on their relationships with others (Uchida et al. [3]). For them, it is important to know what others think of them and how much others know about them to live well. Therefore, items 5 and 7 reflect high independent and low interdependent eudaimonia at the same time, which may explain why they were not associated with self-esteem and life satisfaction for Japanese.

**References**

1. Sotgiu I, Anselmi P, Meneghini AM. Investigating the psychometric properties of the questionnaire for eudaimonic well-being: A Rasch analysis. TPM - Testing, Psychometrics, Methodology in Applied Psychology. 2019; 26: 237-247. doi:10.4473/TPM26.2.5

2. Li J, Fang M, Wang W, Sun G, Cheng Z. The influence of grit on life satisfaction: Self-Esteem as a mediator. Psychologica Belgica, 2018; 58, 51-66. doi.org/10.5334/pb.400

3. Uchida Y, Norasakkunkit V, Kitayama S. Cultural constructions of happiness: Theory and empirical evidence. Journal of Happiness Studies: An Interdisciplinary Forum on Subjective Well-Being, 2004; 5, 223-239. doi.org/10.1007/s10902-004-8785-9
