# Supplementary material for: Reconsidering the structure of the questionnaire for eudaimonic well-being using wide age-range Japanese adult sample: An exploratory analysis
Source: BMC Psychol. 2022 Jan 4;10:3. doi: 10.1186/s40359-021-00707-2 (PMC8729131; doi:10.1186/s40359-021-00707-2)
Supplement: Supplementary file 3 — Additional file 3: The results of ESEM with the items and the factor labels added. [file 40359_2021_707_MOESM3_ESM.docx]

**Additional file3**

APPENDIX Table 5. Standardized factor loadings and residual variances for 10s to 20s with the items and the factor labels added

| 10s to 20s | | | | | | |
| --- | --- | --- | --- | --- | --- | --- |
| Items | | F1 | F2 | F3 | F4 | Residual variance |
| 1 | I find I get intensely involved in many of the things I do each day. | **.687** | .100 | -.129 | .026 | .477 |
| 17 | I find a lot of the things I do are personally expressive for me. | **.611** | .093 | .092 | .069 | .493 |
| 8 | I feel best when I’m doing something worth investing a great deal of effort in. | **.609** | .009 | .158 | .101 | .493 |
| 4 | My life is centered around a set of core beliefs that give meaning to my life. | **.539** | .337 | .031 | -.018 | .435 |
| 7 | Other people usually know better what would be good for me to do than I know myself. ® | **-.516** | .210 | .062 | .232 | .805 |
| 6 | I believe I know what my best potentials are and I try to develop them whenever possible. | **.467** | .391 | .007 | -.017 | .474 |
| 15 | When I engage in activities that involve my best potentials, I have this sense of really being alive. | **.430** | -.022 | .379 | .069 | .562 |
| 14 | I usually know what I should do because some actions just feel right to me. | **.413** | .360 | .127 | .024 | .516 |
| 11 | As yet, I’ve not figured out what to do with my life. ® | .012 | **.651** | -.210 | .212 | .396 |
| 9 | I can say that I have found my purpose in life. | .249 | **.629** | .037 | .012 | .394 |
| 16 | I am confused about what my talents really are. ® | -.048 | **.585** | -.278 | .136 | .523 |
| 21 | I believe I know what I was meant to do in life. | .362 | **.573** | -.035 | -.102 | .402 |
| 2 | I believe I have discovered who I really am. | .293 | **.535** | .000 | -.033 | .503 |
| 10 | If I did not find what I was doing rewarding for me, I do not think I could continue doing it. | .057 | -.017 | **.504** | .002 | .725 |
| 5 | It is more important that I really enjoy what I do than that other people are impressed by it. | .011 | .136 | **.486** | .095 | .735 |
| 3 | I think it would be ideal if things came easily to me in my life. ® | .261 | -.027 | **-.460** | .066 | .780 |
| 18 | It is important to me that I feel fulfilled by the activities that I engage in. | **.427** | -.037 | **.447** | .260 | .387 |
| 13 | I believe it is important to know how what I’m doing fits with purposes worth pursuing. | .246 | .138 | **.429** | -.086 | .664 |
| 12 | I can’t understand why some people want to work so hard on the things that they do. ® | -.025 | .046 | .042 | **.820** | .322 |
| 19 | If something is really difficult, it probably isn’t worth doing. ® | .052 | -.145 | -.062 | **.645** | .587 |
| 20 | I find it hard to get really invested in the things that I do. ® | .163 | .070 | -.341 | **.423** | .627 |
| F1 | Deep and Meaningful Engagement | - |  |  |  |  |
| F2 | Sense of Purpose | .443 | - |  |  |  |
| F3 | Purposeful Personal Expressiveness | .288 | -.060 | - |  |  |
| F4 | Effortful Engagement | .332 | .239 | -.020 |  |  |

」

APPENDIX Table 6. Standardized factor loadings and residual variances for 30s to 40s with the items and the factor labels added.

| 30s to 40s | | | | | |
| --- | --- | --- | --- | --- | --- |
| Items | | F1 | F2 | F3 | Residual variance |
| 21 | I believe I know what I was meant to do in life. | **.814** | .013 | .019 | .329 |
| 9 | I can say that I have found my purpose in life. | **.793** | .013 | -.042 | .368 |
| 11 | As yet, I’ve not figured out what to do with my life. ® | **.772** | -.153 | .262 | .331 |
| 4 | My life is centered around a set of core beliefs that give meaning to my life. | **.748** | .065 | -.104 | .408 |
| 2 | I believe I have discovered who I really am. | **.744** | .009 | -.047 | .446 |
| 6 | I believe I know what my best potentials are and I try to develop them whenever possible. | **.673** | .192 | -.015 | .442 |
| 16 | I am confused about what my talents really are. ® | **.535** | -.264 | .393 | .497 |
| 14 | I usually know what I should do because some actions just feel right to me. | **.492** | .268 | .176 | .591 |
| 1 | I find I get intensely involved in many of the things I do each day. | **.489** | .172 | -.099 | .678 |
| 17 | I find a lot of the things I do are personally expressive for me. | **.468** | .334 | .029 | .587 |
| 3 | I think it would be ideal if things came easily to me in my life. ® | .345 | -.306 | -.007 | .844 |
| 18 | It is important to me that I feel fulfilled by the activities that I engage in. | .016 | **.676** | .218 | .544 |
| 13 | I believe it is important to know how what I’m doing fits with purposes worth pursuing. | .106 | **.615** | -.046 | .564 |
| 15 | When I engage in activities that involve my best potentials, I have this sense of really being alive. | .220 | **.609** | .016 | .512 |
| 10 | If I did not find what I was doing rewarding for me, I do not think I could continue doing it. | -.098 | **.553** | -.071 | .692 |
| 8 | I feel best when I’m doing something worth investing a great deal of effort in. | .270 | **.455** | -.064 | .643 |
| 5 | It is more important that I really enjoy what I do than that other people are impressed by it. | -.094 | **.450** | .126 | .818 |
| 12 | I can’t understand why some people want to work so hard on the things that they do. ® | .004 | .319 | **.664** | .535 |
| 19 | If something is really difficult, it probably isn’t worth doing. ® | -.045 | .196 | **.519** | .736 |
| 20 | I find it hard to get really invested in the things that I do. ® | **.418** | .002 | **.454** | .591 |
| 7 | Other people usually know better what would be good for me to do than I know myself. ® | -.176 | .017 | .344 | .863 |
| F1 | Sense of Purpose | - |  |  |  |
| F2 | Purposeful Personal Expressiveness | .265 | - |  |  |
| F3 | Effortful Engagement | .071 | -.185 |  |  |

APPENDIX Table 7. Standardized factor loadings and residual variances for 50s to 60s with the items and the factor labels added.

| 50s to 60s | | | | | |
| --- | --- | --- | --- | --- | --- |
| Items | | F1 | F2 | F3 | Residual variance |
| 11 | As yet, I’ve not figured out what to do with my life. ® | **.854** | .111 | -.221 | .332 |
| 21 | I believe I know what I was meant to do in life. | **.800** | .035 | -.045 | .639 |
| 9 | I can say that I have found my purpose in life. | **.799** | -.149 | .026 | .355 |
| 2 | I believe I have discovered who I really am. | **.772** | -.060 | -.008 | .419 |
| 4 | My life is centered around a set of core beliefs that give meaning to my life. | **.660** | -.120 | .069 | .528 |
| 16 | I am confused about what my talents really are. ® | **.632** | .308 | -.235 | .497 |
| 6 | I believe I know what my best potentials are and I try to develop them whenever possible. | **.601** | -.020 | .215 | .487 |
| 14 | I usually know what I should do because some actions just feel right to me. | **.543** | .126 | .158 | .579 |
| 20 | I find it hard to get really invested in the things that I do. ® | **.445** | .337 | .020 | .639 |
| 1 | I find I get intensely involved in many of the things I do each day. | **.441** | -.016 | .139 | .736 |
| 7 | Other people usually know better what would be good for me to do than I know myself. ® | -.021 | **.623** | -.044 | .605 |
| 19 | If something is really difficult, it probably isn’t worth doing. ® | -.006 | **.453** | .148 | .792 |
| 12 | I can’t understand why some people want to work so hard on the things that they do. ® | .083 | **.444** | .352 | .680 |
| 18 | It is important to me that I feel fulfilled by the activities that I engage in. | .012 | .228 | **.731** | .451 |
| 13 | I believe it is important to know how what I’m doing fits with purposes worth pursuing. | -.024 | .002 | **.551** | .707 |
| 15 | When I engage in activities that involve my best potentials, I have this sense of really being alive. | .211 | .065 | **.535** | .577 |
| 10 | If I did not find what I was doing rewarding for me, I do not think I could continue doing it. | -.111 | .052 | **.473** | .813 |
| 8 | I feel best when I’m doing something worth investing a great deal of effort in. | .241 | -.099 | **.434** | .652 |
| 17 | I find a lot of the things I do are personally expressive for me. | .395 | -.049 | **.409** | .541 |
| 5 | It is more important that I really enjoy what I do than that other people are impressed by it. | -.044 | .326 | .376 | .802 |
| 3 | I think it would be ideal if things came easily to me in my life. ® | .237 | -.035 | -.248 | .935 |
| F1 | Sense of Purpose | - |  |  |  |
| F2 | Effortful Engagement | .146 | - |  |  |
| F3 | Purposeful Personal Expressiveness | .415 | -.136 |  |  |
